# Enhancing the Laccase Production and Laccase Gene Expression in the White-Rot Fungus *Trametes velutina* 5930 with Great Potential for Biotechnological Applications by Different Metal Ions and Aromatic Compounds

**DOI:** 10.1371/journal.pone.0079307

**Published:** 2013-11-11

**Authors:** Yang Yang, Fuxiang Wei, Rui Zhuo, Fangfang Fan, Huahua Liu, Chen Zhang, Li Ma, Mulan Jiang, Xiaoyu Zhang

**Affiliations:** 1 College of Life Science and Technology, Huazhong University of Science and Technology, Wuhan, China; 2 Key Laboratory of Oil Crops Biology of Ministry of Agriculture in China, Oil Crops Research Institute of Chinese Academy of Agricultural Sciences, Wuhan, China; Missouri University of Science and Technology, United States of America

## Abstract

Laccase is useful for various biotechnological and industrial applications. The white-rot fungus *Trametes velutina* 5930 and its laccase, isolated from the Shennongjia Nature Reserve in China by our laboratory, has great potential for practical application in environmental biotechnology. However, the original level of laccase produced by *Trametes velutina* 5930 was relatively low in the absence of any inducer. Therefore, in order to enhance the laccase production by *Trametes velutina* 5930 and make better use of this fungus in the field of environmental biotechnology, the regulation of laccase production and laccase gene expression in *Trametes velutina* 5930 were investigated in this study. Different metal ions such as Cu^2+^ and Fe^2+^ could stimulate the laccase synthesis and laccase gene transcription in *Trametes velutina* 5930. Some aromatic compounds structurally related to lignin, such as tannic acid, syringic acid, cinnamic acid, gallic acid and guaiacol, could also enhance the level of laccase activity and laccase gene transcription. We also found that there existed a positive synergistic effect of aromatic compound and metal ion on the laccase production and laccase gene transcription in *Trametes velutina* 5930. Taken together, our study may contribute to the improvement of laccase productivity by *Trametes velutina* 5930.

## Introduction

White-rot fungi are unique in their strong ability to extensively degrade lignin and mineralize a variety of recalcitrant synthetic chemicals similar to lignin in structure. Due to its capability of biodegrading xenobiotics and recalcitrant pollutants, white-rot fungi have been widely applied in bioremediation and environmental biotechnology [1], [2]. White-rot fungi, which are mostly basidiomycetes, can secrete extracellular and non-specific lignin modifying enzymes (LMEs) that play an important role in the degradation of lignin and xenobiotics. The ligninolytic enzymes of white-rot fungi mainly comprise of lignin peroxidase (LiP), manganese dependent peroxidase (MnP), and laccase [1], [3].

Laccase (EC 1.10.3.2) belongs to a group of copper-containing polyphenol oxidases that can catalyze the four-electron reduction of O_2_ to H_2_O coupled with the oxidation of phenolic compounds. As a ligninolytic enzyme produced by white-rot fungi, laccase exhibits a broad substrate specificity and unique ability of biodegradation [4]. Laccase has been widely applied in many fields, such as delignification of lignocellulosic biomass, detoxification of recalcitrant pollutants, decolorization of industrial dyes and textile dye effluents, biological bleaching in pulp and paper industries, juice and wine clarification, and biosensors [5], [6].

The great potential and value in industrial and biotechnological applications have resulted in a strong interest in obtaining a large amount of laccase for practical use. However, the native production of laccase by white-rot fungi is relatively very low and cannot meet the demand of practical applications in industry and biotechnology [7]. Therefore, many efforts have been concentrated on increasing the laccase production by inducing the laccase gene expression in white-rot fungi. Study on the regulation of laccase gene expression may greatly contribute to the improvement of native laccase productivity in white-rot fungi [7], [8]. Previous research has suggested that the synthesis of laccase and the expression of laccase gene can be stimulated by some different external factors, such as metal ions [9]–[12], aromatic compounds structurally related to lignin or lignin derivatives [13]–[17], nutrient nitrogen [9], [18] and carbon [11], [19]. The regulation of laccase gene expression by these factors mainly occurs at the level of transcription [7], [8]. The effect of different factors on the transcription of the same laccase gene is different [15]. The effect of the same factor on the transcription of different laccase genes encoding various isoenzymes is also very different, with some being constitutively expressed and others being inducible [11]. The putative *cis*-acting responsive elements present in the promoter of laccase gene, such as metal-responsive elements, xenobiotic-responsive elements may be involved in the transcriptional regulation of laccase gene [12], [18], [19], [20].

In our previous work [21], a new white-rot fungi strain *Trametes* sp. 5930, which was isolated from the virgin forest of Shennongjia Nature Reserve in China, has promising potential in applications in the field of environmental biotechnology. Laccase played a very important role in the decolorization of different dyes by this fungus. The laccase gene, *lac5930-1*, and its corresponding full-length cDNA were cloned and characterized from *Trametes* sp. 5930. Based on morphology and analysis of internal transcribed spacer (ITS) sequence of ribosome DNA, this fungal strain was identified as *Trametes velutina* 5930 [21]. However, the original level of laccase produced by *Trametes velutina* 5930 is relatively low in the absence of any inducer. Therefore, in order to enhance the laccase production by *Trametes velutina* 5930, we here studied the induction of laccase activity and regulation of laccase gene expression in *Trametes velutina* 5930 by different factors. In this study, we found that different metal ions as well as the aromatic compounds with different structure could stimulate the laccase gene transcription and laccase synthesis in *Trametes velutina* 5930. There existed a synergistic effect of metal ion and aromatic compound on the induction of laccase production and laccase gene transcription.

## Materials and Methods

### Strain, Medium and Culture Conditions

*Trametes velutina* 5930 was isolated from the virgin forest of Shennongjia Nature Reserve in China in our previous work [21] and preserved in Institute of Environment & Resource Microbiology, Huazhong University of Science and Technology, Wuhan, China. *Trametes velutina* 5930 was maintained on potato-dextrose agar (PDA) medium. The basal GYP medium used for cultures contained 20 g glucose/l, 5 g yeast extract/l, 5 g peptone from casein/l, and 1 g MgSO4·7H2O/l. The pH was adjusted to 5.0 with H_3_PO_4_ prior to sterilization [12]. Five agar plugs obtained from the outer circumference of a fungal colony growing on potato dextrose plates were inoculated into 100 ml volumes of GYP medium in 250 ml Erlenmeyer flasks. The fungus was grown at 28°C with shaking at 150 rpm for 7 days. Previous research has suggested that the laccase synthesis and laccase gene transcription in the white-rot fungus *Coriolopsis gallica* could be significantly induced when the aromatic compound tannic acid was added in 7-day-old cultures [22]. Therefore, in order to better compare our results with those of previous research [22], the following inducers were added into the actively growing 7-day-old cultures of *Trametes velutina* 5930 at various final concentrations: CuSO_4_·5H_2_O (0.5, 1 mM), FeSO_4_·7H_2_O (0.04, 0.2 mM), tannic acid (0.2 mM), cinnamic acid (2 mM), syringic acid (1 mM), gallic acid (0.1 mM), and guaiacol (1 mM). To study the synergistic effect of copper and aromatic compound, 0.5 mM CuSO_4_·5H_2_O and 1 mM syringic acid were simultaneously added into the 7-day-old cultures of *Trametes velutina* 5930. After adding the inducers, the fungal cultures were then grown at 28°C with shaking at 150 rpm continuously. Samples from triplicate flasks were withdrawn at different time intervals, centrifuged, and the clear supernatant was used for measuring the enzyme activity. A reference culture without any inducer served as control. All experiments were performed in triplicate.

### Enzyme Activity Assay

Laccase activity was determined with 2,2′-azino-bis (3-ethylbenzthiazoline-6-sulfonate) (ABTS) as the substrate [9]. Assay mixtures contained 0.5 mM ABTS, 0.1 M sodium acetate (pH = 5.0) and 100 µl culture fluid. Oxidation of ABTS was monitored by determining the increase in *A*_420_ (ε = 36,000 M^−1^ cm^−1^). One unit of enzyme activity was defined as the amount of enzyme required to oxidize 1 µmol of ABTS per min [9].

### Determination of Mycelial Dry Weight

Determination of mycelial dry weight was performed according to the method described by Manubens et al. [23]. Triplicate flasks were harvested and filtered through Whatman No. 1 paper that had previously been dried at 100°C to a constant weight. The mycelium retained on the filter paper was dried at 100°C to a constant weight and the mycelial weight was determined [23].

### Extraction of RNA

At the time of harvesting, fungal mycelia were collected by filtration, washed twice with distilled water, and immediately frozen in liquid nitrogen. The frozen mycelia were ground to a powder with a mortar and pestle. Then total RNA was extracted using TRIZOL reagent (Invitrogen) according to the manufacturer’s instructions, followed by RNase-free DNase (Promega) digestion to remove the genomic DNA (gDNA) contamination.

### Quantitative real-time RT-PCR (qRT-PCR) for Measuring the Transcription level of Laccase gene *lac5930-1* in *Trametes Velutina* 5930 Subjected to Different Inducers

Total RNA was used as the template to generate first-strand cDNA. First-strand complementary DNA (cDNA) was then synthesized using a PrimeScript RT Reagent Kit with gDNA Eraser (TAKARA). Two microliters of RT product was used as a template for qRT-PCR. qRT-PCR was performed using an iCycler iQ5 real-time PCR system (Bio-Rad) and SYBR *Premix Ex Taq* II Kit (Tli RNaseH Plus) (TAKARA) according to the manufacturer’s instructions. The specific primer pairs used for quantitative measurement of the transcription of *lac5930-1* gene were listed as follows: qRT-lac5930-Fw (5′-agcgcttcagcttcttcgttac-3′) and qRT-lac5930-Rv (5′-ccagtggatactggtggacttg-3′). They were designed according to the 1563 bp full-length cDNA of laccase gene cloned in our previous work [21] (GenBank Accession No. GU738021). The qRT-PCR mixture (25 µl) contained 2.0 µl of cDNA and 0.4 µM of each gene-specific primer as well as 1× SYBR *Premix Ex Taq* II (TAKARA). The qRT-PCR was performed as follows: 10 min at 95°C followed by 30 cycles of 30 s at 95°C, 30 s at 55°C and 30 s at 72°C, followed by a melting cycle from 55°C to 95°C to check for amplification specificity. The *gpd* gene encoding glyceraldehyde-3-phosphate dehydrogenase was used as internal control. The primers for amplification of the specific 152 bp fragment of *gpd* gene were qRT-gpd-Fw (5′-aagaaggtcgttatctccgctcc-3′) and qRT-gpd-Rv (5′-ttgtcgttgatgaccttggcg-3′). The relative abundance of mRNAs was normalized against the levels of *gpd*. Each sample was amplified in triplicate in each experiment. Two independent experiments were performed, and they showed the same results.

### Cloning of the 5′-flanking Sequence Upstream of the Start Codon of *lac5930-1* Gene by Self-Formed Adaptor PCR (SEFA PCR)

In order to obtain the promoter region of *lac5930-1* gene, Self-Formed Adaptor PCR (SEFA PCR) [24] was performed to amplify the 5′-flanking sequence upstream of start codon of *lac5930-1* gene. The nested primers (lac-5-SP1∶5-gtgatgagcggggaggggaacacgccgttg-3,lac-5-SP2∶5-gggcgtccgtgacgacgaggttcgctgacg-3,lac-5-SP3∶5-ggtaacgaagaagctgaagcNNNNNNNNNNcaccat-3, and lac-5-SP4∶5-gaggagggagatagcggagacggagaaccc-3) were designed based on the known structural gene sequence of *lac5930-1*. lac-5-SP1, lac-5-SP2 and lac-5-SP4 were gene-specifi c primers and had high annealing temperatures (about 70°C). lac-5-SP3 was a partially degenerate primer. The putative *cis*-acting elements in the promoter region of laccase gene were predicted and identified with SoftBerry-NSITE/Recognition of Regulatory motifs(http://www.softberry.ru/berry.phtml?topic=nsite&group=programs&subgroup=promoter). The putative *cis*-acting elements in the promoter region of laccase gene were also examined using the MatInspector software (http://www.genomatix.de/products/MatInspector/).

## Results

### Cloning and Analysis of the Promoter Region of *lac5930-1* gene from *Trametes Velutina* 5930

**Figure 1:**
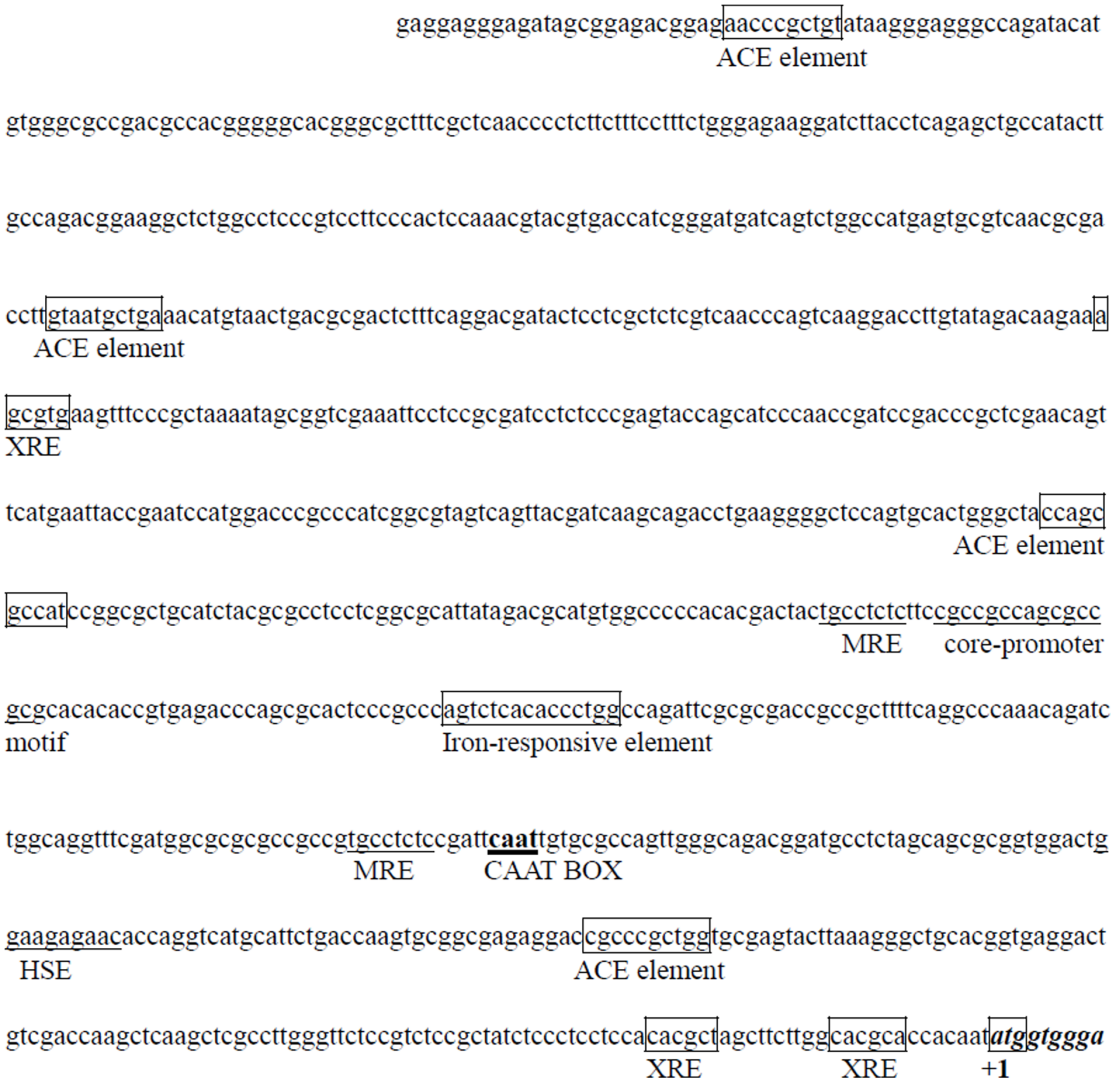
The 972′-flanking sequence upstream of the start codon ATG in *lac5930-1* gene encoding laccase. The putative *cis-*acting responsive elements in the promoter region of this gene were underlined or boxed. MRE: metal-responsive elements; XRE: xenobiotic-responsive elements; HSE: heat-shock element; ACE: ACE element; CAAT box and a core-promoter-motif GC box were also underlined. The first nucleotide of the start codon ATG was designated as +1.

We have previously cloned and characterized a laccase gene, *lac5930-1*, and its corresponding full-length cDNA from white-rot fungus *Trametes velutina* 5930 isolated from Shennongjia Nature Reserve in China [21]. Here, in order to study the regulation of laccase synthesis and *lac5930-1* gene transcription in *Trametes velutina* 5930, the 972 bp 5′-flanking sequence upstream of the start codon ATG in *lac5930-1* gene was first obtained and analyzed for the presence of putative *cis*-acting elements involved in transcriptional regulation. The putative promoter region of *lac5930-1* extending 972 bp upstream of the start codon was shown in Fig. 1. A CAAT box and a core-promoter-motif GC box were located at nt positions −226 and −371 upstream from the start codon ATG respectively. Some putative *cis*-acting responsive elements that may be involved in the regulation of *lac5930-1* gene transcription were also found in the promoter region (Fig. 1). Two putative metal-responsive elements (MREs) adhering to the consensus sequence TGCRCNC, which confers the ability to respond to heavy metals [25], were present at positions −239 and −382, respectively. Three putative xenobiotic-responsive elements (XREs) matching the consensus sequence CACGCW [26] were located at positions −13, −29 and −636. Four ACE elements adhering to the consensus sequence HWHNNGCTGD or NTNNHGCTGN [27] were present at positions −128, −455, −726 and −947, respectively. Previous research has suggested that ACE element was the target sequence of the ACE1 copper-responsive transcription factor originally found in *Saccharomyces cerevisiae* [28]. Recent research has found that expression of gene encoding laccase in the fungus *Ceriporiopsis subvermispora* was mediated by an ACE1-like copper-fist transcription factor that can interact specifically with the ACE element from the promoter of laccase gene [20]. One potential heat-shock element (HSE) composed of the repeated 5 bp NGAAN motif [29] was located at position −176 relative to start codon ATG. One putative iron-responsive element [30] was located at position −323.

The presence of some special *cis*-acting responsive elements (MRE, XRE, ACE) in the promoter region of *lac5930-1* gene implied that the transcription of *lac5930-1* gene may be regulated by metal ions, xenobiotics, and aromatic compounds. In order to validate this hypothesis as well as effectively improve the laccase production by *Trametes velutina* 5930, the effects of different metal ions and aromatic compounds on the laccase production and laccase gene transcription were evaluated further in the following work.

### Effect of Different Metal Ions on the Extracellular Laccase Production and Laccase Gene Transcription in *Trametes Velutina* 5930

**Figure 2:**
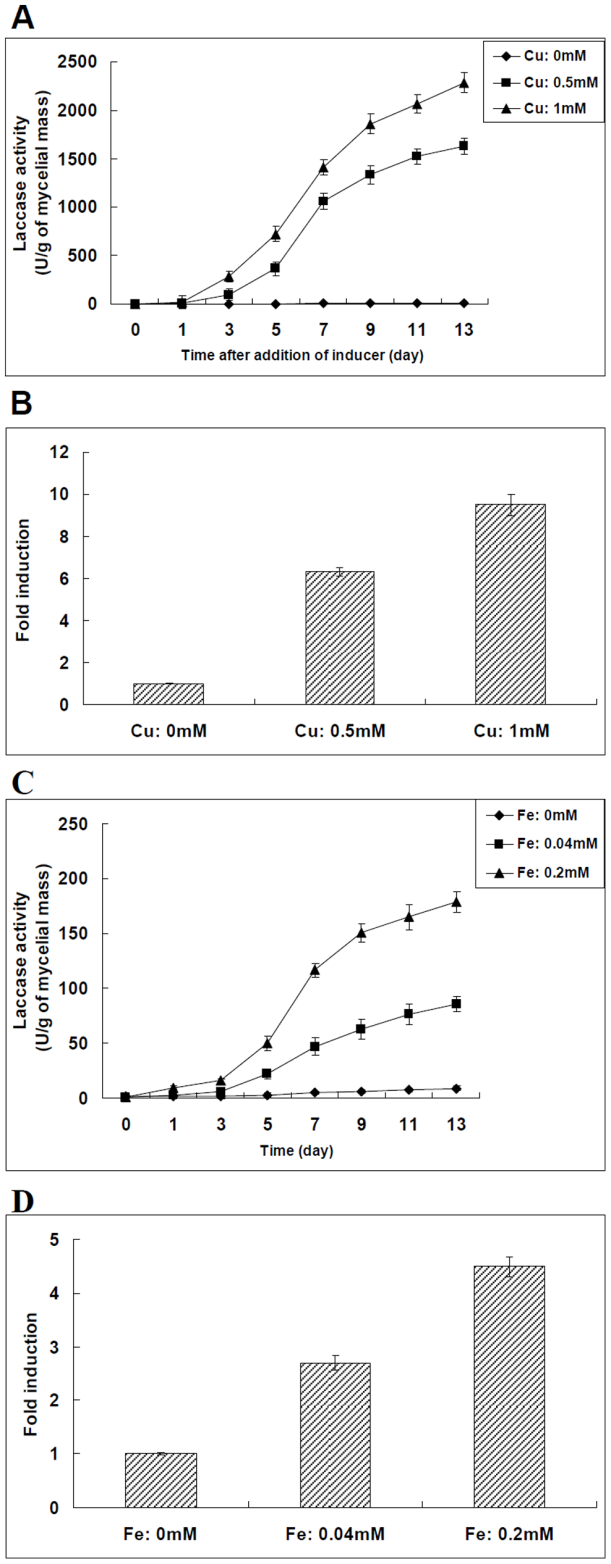
Effect of different metal ions on the extracellular laccase production and laccase gene transcription in *Trametes velutina* 5930. (A): Time course of laccase activity in the extracellular fluid of *Trametes velutina* 5930 grown in the presence of different concentrations of Cu^2+^ (0, 0.5, 1 mM). (B): Comparison of the transcription level of laccase gene-*lac5930-1* in *Trametes velutina* 5930 grown in the medium supplemented with different concentrations of Cu^2+^ (0, 0.5, 1 mM) for 13 days. The transcription level of *lac5930-1* gene in *Trametes velutina* 5930 exposed to 0 mM Cu^2+^ is set as 1-fold. Error bars represent standard deviation. (C): Time course of laccase activity in the extracellular fluid of *Trametes velutina* 5930 grown in the presence of different concentrations of Fe^2+^ (0, 0.04, 0.2 mM). (D): Comparison of the transcription level of laccase gene-*lac5930-1* in *Trametes velutina* 5930 grown in the medium supplemented with different concentrations of Fe^2+^ (0, 0.04, 0.2 mM) for 13 days. The transcription level of *lac5930-1* gene in *Trametes velutina* 5930 exposed to 0 mM Fe^2+^ is set as 1-fold. Error bars represent standard deviation.

The 7-day-old cultures were treated with different concentrations of metal ions including Cu^2+^ and Fe^2+^. The extracellular activities of laccase produced by *Trametes velutina* 5930 were measured at various times thereafter. As shown in Fig. 2A and C, the extracellular laccase activities remained very low throughout the incubation in the absence of any inducer. However, the extracellular laccase production could be significantly stimulated by Cu^2+^ and Fe^2+^. For example, the extracellular laccase activity of *Trametes velutina* 5930 in the absence of inducer for 13 days was found to be only 8.9 U/g of mycelial mass, but the extracellular laccase activities obtained after 13 days of incubation in the presence of 1 mM Cu^2+^ and 0.2 mM Fe^2+^ were increased to be 2280 and 178 U/g of mycelial mass, respectively (Fig. 2A and C). We also observed that the extracellular laccase activity increased as the concentration of exogenous metal ions increased.

At the end of incubation, the transcription levels of laccase gene-*lac5930-1* in cultures treated with different concentrations of metal ions were determined. As shown in Fig. 2B and D, the different metal ions including Cu^2+^ and Fe^2+^ could stimulate the transcription of *lac5930-1* gene in *Trametes velutina* 5930. The transcription levels of *lac5930-1* gene in *Trametes velutina* 5930 subjected to 0.5 and 1 mM Cu^2+^ for 13 days were increased to about 6.3 and 9.5 times that of the same strain subjected to 0 mM Cu^2+^ for the same amount of time (Fig. 2B). The transcription levels of *lac5930-1* gene in cultures grown in the presence of 0.04 and 0.2 mM Fe^2+^ for 13 days were about 2.7 and 4.5-fold greater than that in cultures grown in the absence of Fe^2+^ (Fig. 2D).

### Effect of Different Aromatic Compounds on the Extracellular Laccase Production and Laccase Gene Transcription in *Trametes Velutina* 5930

**Figure 3:**
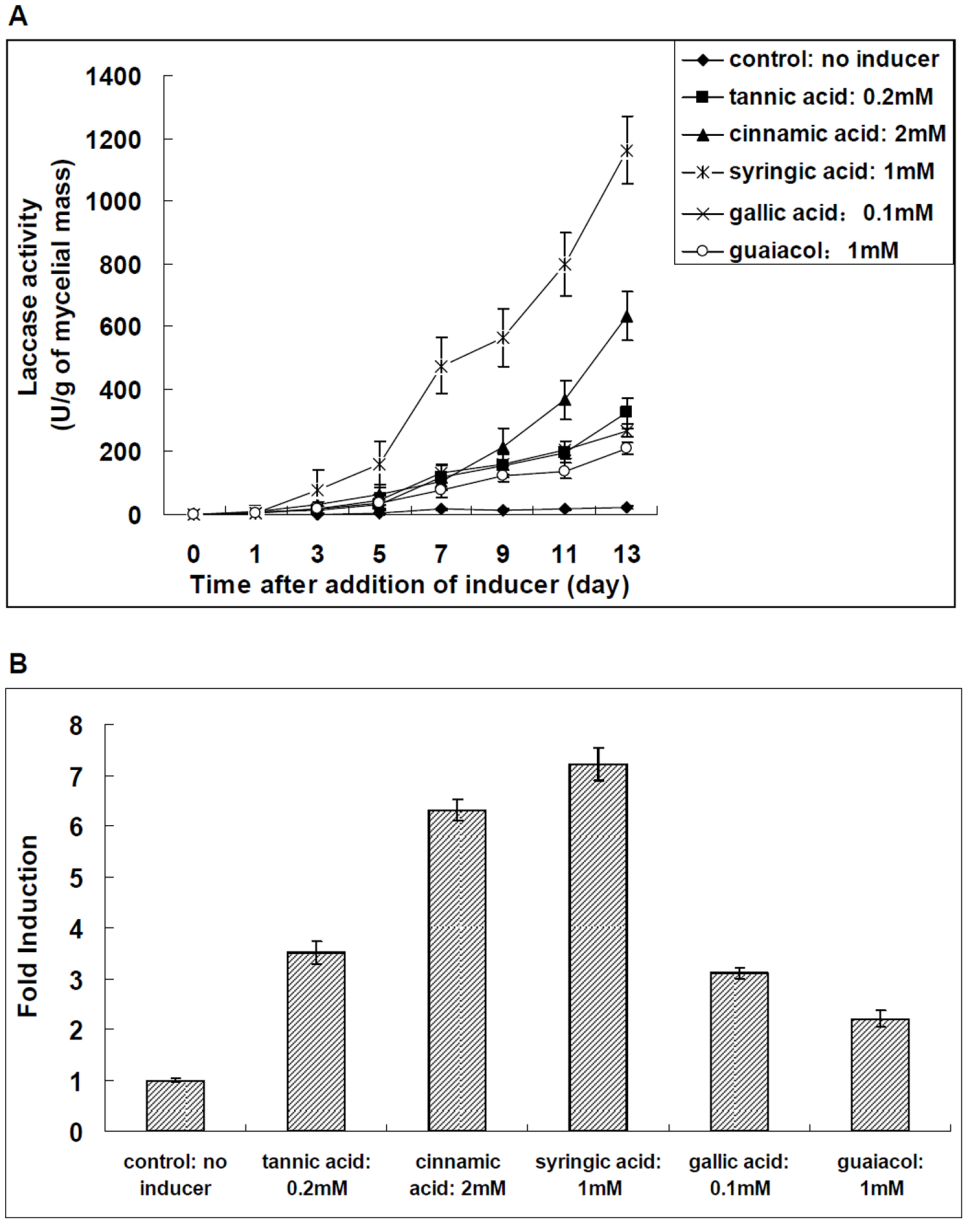
Effect of different aromatic compounds on the extracellular laccase production and laccase gene transcription in *Trametes velutina* 5930. (A): Time course of laccase activity in extracellular fluid of *Trametes velutina* 5930 culture after addition of different concentrations of aromatic compounds such as tannic acid (0.2 mM), cinnamic acid (2 mM), syringic acid (1 mM), gallic acid (0.1 mM) and guaiacol (1 mM). (B): Relative induction of the transcription of *lac5930-1* gene under different concentrations of aromatic compounds. The transcription level of *lac5930-1* gene in *Trametes velutina* 5930 without addition of any inducer was set as 1-fold (control). Error bars represent standard deviation.

The effects of different aromatic compounds, including tannic acid, syringic acid, cinnamic acid, gallic acid and guaiacol, on the extracellular laccase production and laccase gene transcription in *Trametes velutina* 5930 were also determined. Laccase production by *Trametes velutina* 5930 in response to different aromatic compounds were shown in Fig. 3A. A significant increase in the laccase synthesis was observed when different aromatic compounds were added into the cultures. After 13 days of incubation in the presence of various inducers, the extracellular laccase activity increased to 325 U/g of mycelial mass (0.2 mM tannic acid, Fig. 3A), 675 U/g of mycelial mass (2 mM cinnamic acid, Fig. 3A), and 1160 U/g of mycelial mass (1 mM syringic acid, Fig. 3A). When 0.1 mM gallic acid and 1 mM guaiacol was added into the actively growing cultures, the highest laccase activity was detected to be 268 U/g of mycelial mass (0.1 mM gallic acid, Fig. 3A) and 210 U/g of mycelial mass (1 mM guaiacol, Fig. 3A) at 13 days of growth, respectively. However, very low enzyme activity was detected in cultures not supplemented with any aromatic compound.

At the end of incubation, the transcription levels of laccase gene-*lac5930-1* in cultures supplemented with different aromatic compounds were also measured. We found that the transcription of *lac5930-1* gene in *Trametes velutina* 5930 could be induced by all of the aromatic compounds tested in this study. As shown in Fig. 3B, tannic acid (0.2 mM) increased the transcription level of *lac5930-1* gene approximately 3.5-fold, compared to the control not treated with tannic acid. The transcription level of *lac5930-1* gene induced by 2 mM cinnamic acid, 1 mM syringic acid, 0.1 mM gallic acid and 1 mM guaiacol resulted in an increase of 6.3, 7.2, 3.1 and 2.2-fold, respectively, compared to the control without addition of any aromatic compound (Fig. 3B).

**Figure 4:**
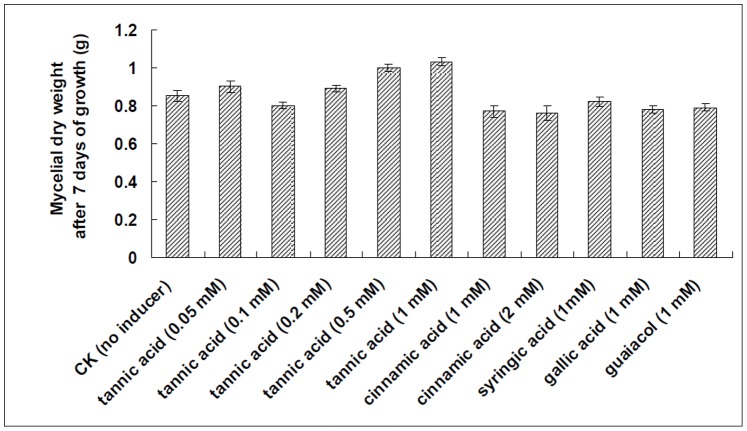
Tolerance of *Trametes velutina* 5930 to the aromatic compounds including tannic acid, cinnamic acid, syringic acid, gallic acid and guaiacol. The various concentrations of aromatic compounds were added to 100

In order to determine the toxicity of different aromatic compounds to the growth of *Trametes velutina* 5930, the various concentrations of aromatic compounds were added to the medium at the time of inoculation, then the mycelial dry weight of cultures after 7 days of growth were measured. As shown in Fig. 4, the aromatic compounds tested in this study did not inhibit the growth of *Trametes velutina* 5930 in submerged cultures.

### Synergistic Action of Metal Ion and Aromatic Compound for Inducing the Laccase Production and Laccase gene Transcription in *Trametes Velutina* 5930

The effect of simultaneous use of copper and aromatic compound on the laccase production and laccase gene transcription was further evaluated. Our results suggested that there existed a positive synergistic effect of aromatic compound and copper on the laccase production and laccase gene transcription in *Trametes velutina* 5930.

**Figure 5:**
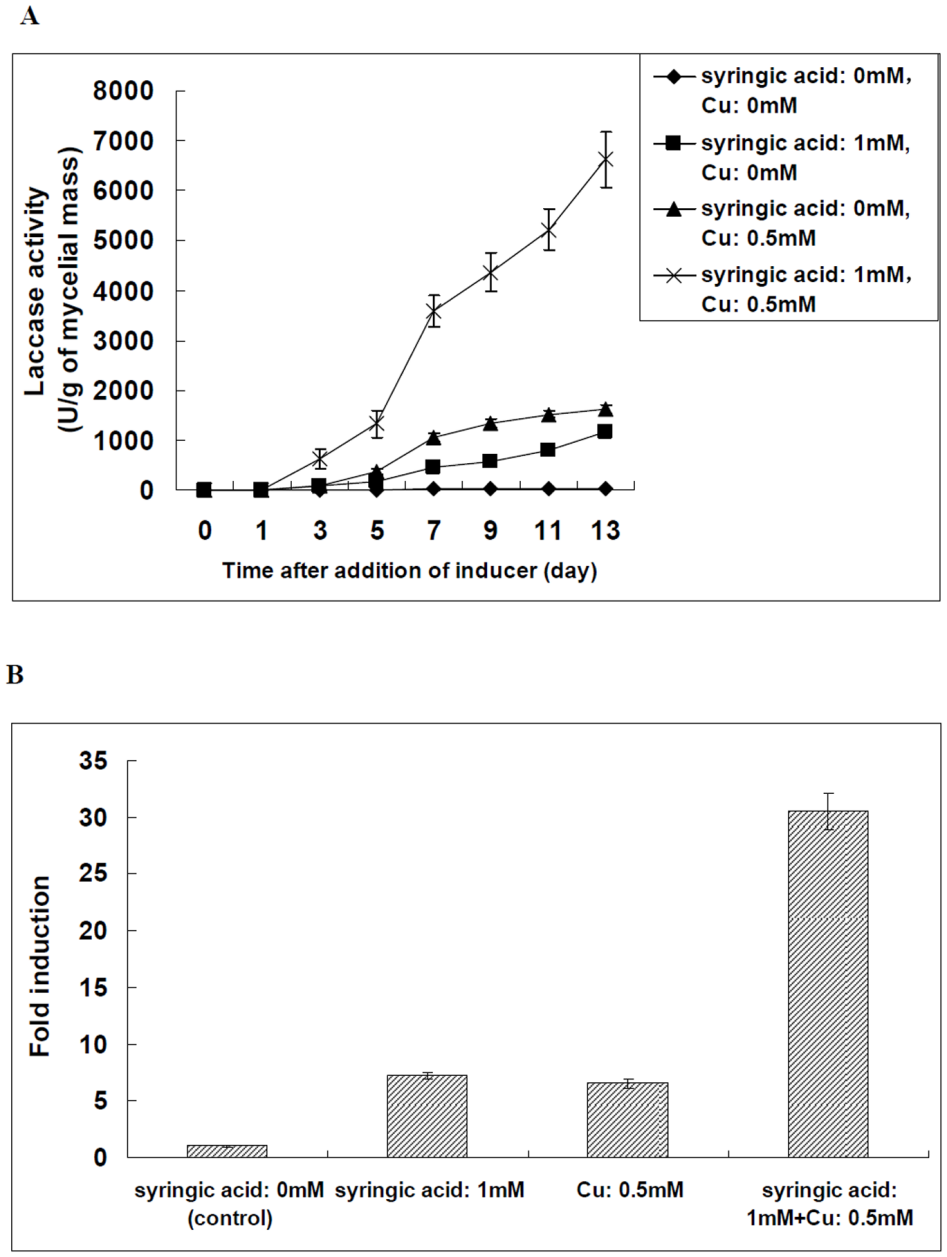
Effect of simultaneous addition of metal ion and aromatic compound on the extracellular laccase production and laccase gene transcription in *Trametes velutina* 5930. (A): Time course of laccase activity in extracellular fluid of *Trametes velutina* 5930 culture after addition of syringic acid (1 mM) and Cu^2+^ (0.5 mM) simultaneously. (B): Relative induction of the transcription of *lac5930-1* gene when the cultures were supplemented with syringic acid (1 mM) and Cu^2+^ (0.5 mM) simultaneously. The transcription level of *lac5930-1* gene in *Trametes velutina* 5930 exposed to 0 mM syringic acid and Cu^2+^ was set as 1-fold (control). Error bars represent standard deviation.

As shown in Fig. 5A, the higher laccase activity (6630 U/g of mycelial mass) was obtained after 13 days of cultivation when syringic acid (1 mM) and Cu^2+^ (0.5 mM) were added into the liquid culture of *Trametes velutina* 5930 simultaneously (Fig. 5A). The laccase activity obtained with the simultaneous addition of copper and aromatic compound was higher than that obtained with individual inducer (Fig. 5A).

The effect of simultaneous use of copper and aromatic compound on the laccase gene transcription was also evaluated. As shown in Fig. 5B, simultaneous addition of 1 mM syringic acid and 0.5 mM Cu^2+^ increased the level of *lac5930-1* gene transcription approximately 30.5-fold, compared to the control not treated with any inducer (Fig. 5B).

**Figure 6:**
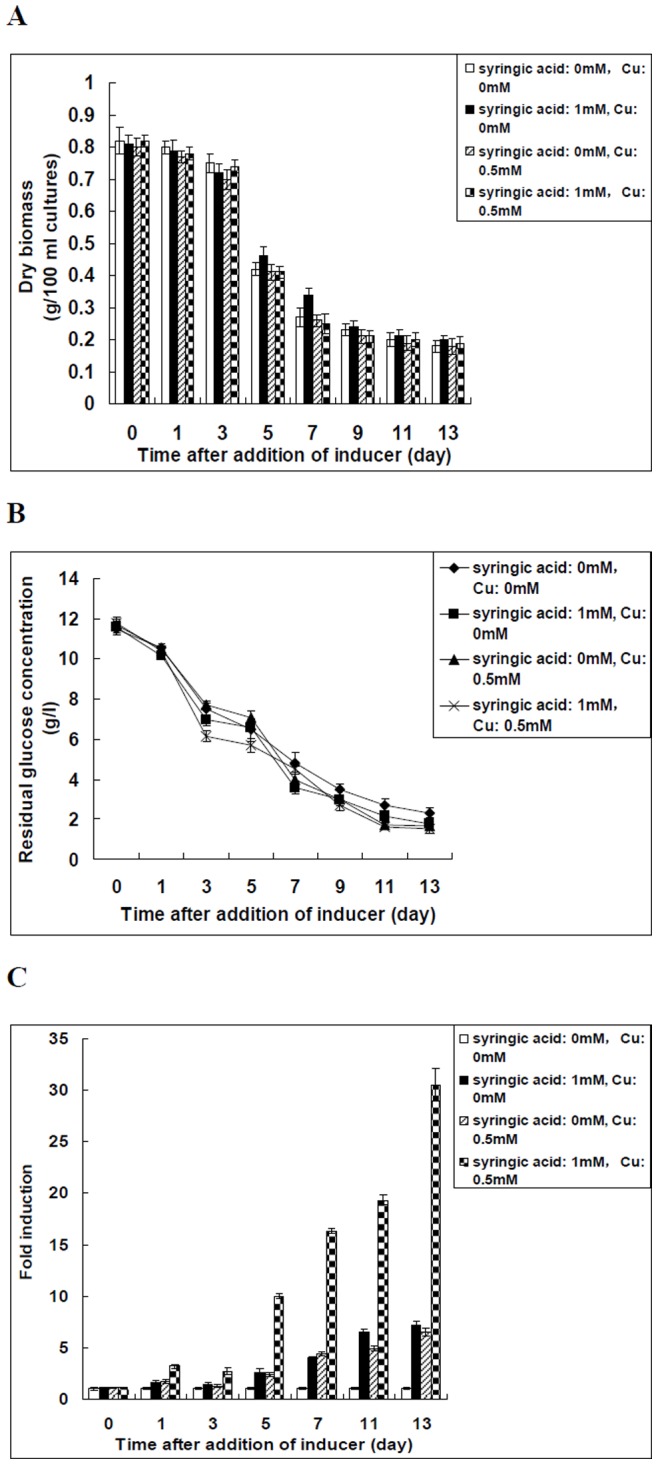
Measurement of the dry biomass at the time points of sampling (A), residual glucose concentration in the medium (B), as well as the transcription level of laccase gene over time (C) after syringic acid and Cu^2+^ were added into the cultures simultaneously or alone. The transcription level of *lac5930-1* gene in *Trametes velutina* 5930 exposed to 0 mM syringic acid and Cu^2+^ at each time point was set as 1-fold (control). Error bars represent standard deviation.

Based on our results, simultaneous addition of syringic acid and Cu^2+^ was the optimised condition for enhancing the laccase expression in *Trametes velutina* 5930. Additional experimental data and a more complete analysis of the cultivation were obtained for this optimised condition. After syringic acid and Cu^2+^ were added into the cultures simultaneously, the dry biomass at the time points of sampling, residual glucose concentration in the medium, as well as the transcript level of laccase gene over time were measured and shown in Fig. 6. The biomass yield and residual glucose concentration of all of the cultures (including the control without addition of any inducer) gradually decreased with time after addition of inducers (Fig. 6).

## Discussion

In this work, it is found that the laccase gene transcription in *Trametes velutina* 5930 can be induced by metal ions such as Cu^2+^ and Fe^2+^ (Fig. 2). Two putative metal-responsive elements (MREs) and four ACE elements which exist in the promoter region of *lac5930-1* gene (Fig. 1) may be involved in the induction of *lac5930-1* gene transcription by these metal ions. Our work also suggests that the laccase gene transcription in *Trametes velutina* 5930 can be enhanced by different aromatic compounds. Three putative xenobiotic-responsive elements (XREs) are found in the promoter region of *lac5930-1* gene (Fig. 1). Previous research has demonstrated that XRE is the functional *cis*-acting element which plays an important role in regulating the eukaryotic gene transcription by aromatic compounds [26]. Thus, the putative xenobiotic-responsive elements (XREs) present in the promoter region of *lac5930-1* gene may function as the regulatory element taking part in the regulation of laccase gene transcription by aromatic compounds. Taken together, some putative *cis*-acting elements present in the promoter region of *lac5930-1* gene (metal-responsive elements, xenobiotic-responsive elements and ACE elements) may be closely related to the induction of laccase gene transcription by different factors. However, the precise mechanisms underlying the correlation between these putative *cis*-elements and the induction effects of different factors remain unclear. Research into the mechanism of regulating the laccase gene transcription through these putative *cis-*acting elements is under way in our laboratory.

To our knowledge, there have been few reports on the effect of tannic acid and cinnamic acid on the laccase production and laccase gene expression in white-rot fungi [22], [31], [32]. Our study indicates that tannic acid and cinnamic acid can function as the promising inducers for improving the yield of laccase.

As shown in Fig. 3B, the laccase gene transcription level induced by cinnamic acid (2 mM) and syringic acid (1 mM) treatment was similar, but the measured laccase activities were different under the same condition (Fig. 3A). The laccase activity induced by 1 mM syringic acid was higher than that induced by 2 mM cinnamic acid (Fig. 3A). This difference might be due to the possibility that aromatic compounds may stimulate the laccase synthesis by other ways besides inducing the laccase gene transcription. That is, not only the regulation of gene transcription but also other mechanisms may be involved in the stimulation of laccase activity by aromatic compounds. For example, laccase produced by white rot fungi is often secreted extracellularly as different isozymes. Previous research has suggested that different aromatic compounds can selectively induce the production of different laccase isozymes [8]. The different types of laccase isozymes may result in the different laccase activity. Furthermore, addition of some aromatic compounds may decrease the extracellular proteolytic activity, thus increasing the laccase stability. That is, some aromatic compounds may enhance the laccase activity by contributing to the enzyme stabilization. Thus, we speculate that the higher laccase activity induced by syringic acid may be due to not only the increase of laccase gene transcription, but also the induction of different laccase isozymes and improvement of the enzyme stability. We are currently attempting to verify this hypothesis in our laboratory.

Although our study has suggested that the laccase production can be enhanced by different metal ions and aromatic compounds, laccase production by *Trametes velutina* 5930 is still low if compared with previous works [10]. Thus, further studies need to be performed to improve the laccase production of *Trametes velutina* 5930 to a higher degree by optimizing conditions.

## Conclusions

Laccase production and laccase gene transcription in *Trametes velutina* 5930 can be stimulated by a range of factors including different metal ions and aromatic compounds which are structurally related to lignin. There exists a positive synergistic effect of metal ion and aromatic compound on the laccase production and laccase gene transcription in *Trametes velutina* 5930. Our study may effectively contribute to the improvement of laccase productivity by *Trametes velutina* 5930.
